# PGC-1α and PPARs cooperatively mediate photoreceptor neuroprotection in *rd1* mouse inherited retinal degeneration

**DOI:** 10.1038/s41419-026-09254-3

**Published:** 2026-09-14

**Authors:** Lan Wang, Jie Yan, Javier Sancho-Pelluz, Qianlu Yang, Zhulin Hu, Kangwei Jiao, Mathias W. Seeliger, François Paquet-Durand

**Affiliations:** 1 Cell Death Mechanism Group, Institute for Ophthalmic Research, University of Tübingen, Tübingen, Germany; 2 Graduate Training Centre of Neuroscience, University of Tuebingen, Tübingen, Germany; 3 Yunnan Eye Institute & Key Laboratory of Yunnan Province, Yunnan Eye Disease Clinical Medical Center, Affiliated Hospital of Yunnan University, Yunnan University, Kunming, China; 4 Departamento de Anatomía y Fisiología, Facultad de Medicina y Ciencias de la Salud, Universidad Católica de Valencia San Vicente Mártir, Valencia, Spain; 5 Department of breast surgery of The Third Affiliated Hospital of Kunming Medical University, Yunnan Cancer Hospital, Peking University Cancer Hospital Yunnan, Kunming, China; 6 Division of Ocular Neurodegeneration, Institute for Ophthalmic Research, University of Tübingen, Tübingen, Germany

**Keywords:** Cell death in the nervous system, Retina, Metabolic pathways

## Abstract

Retinitis pigmentosa (RP) is a group of inherited diseases characterized by primary rod photoreceptor dysfunction and progressive cone cell death. Due to their very high energy demand, the degeneration of photoreceptors may be linked to insufficient energy supply or metabolic imbalance. Critical transcription factors that regulate metabolism, such as peroxisome proliferator-activated receptors (PPARs) and PPAR gamma coactivator 1α (PGC-1α), have been found to play important roles in neurodegenerative diseases, but their potential roles in RP have yet not been elucidated. Here, organotypic retinal explant cultures derived from retinal degeneration 1 (*rd1*) mice were used to investigate the effects of PPARα, PPARγ, PPARβ/δ agonists, as well as PGC-1α activation and inhibition. Photoreceptor death was quantified using the TUNEL assay, while in situ activity assays were used to monitor effects of PPARs and PGC-1α on poly (ADP-ribose) polymerase (PARP) and calpain activity. Generation of poly (ADP-ribose) (PAR) and activation of calpain-1 and calpain-2 were evaluated by immunostaining. PGC-1α/PPAR-associated transcriptional changes were assessed by RT-qPCR. We found that PPARβ/δ agonists had limited effects, while treatment targeting PPARα, PPARγ, and PGC-1α significantly reduced photoreceptor death and PARP activity in *rd1* retina. RT-qPCR analysis in treated *rd1* retina confirmed upregulation of genes downstream of PGC-1α/PPAR signaling. Stimulation of the histone deacetylase sirtuin-1, an upstream regulator of PGC-1α, had no beneficial effect on photoreceptor viability unless combined with PARP inhibition. Furthermore, PPARγ and PGC-1α effectively suppressed overall calpain activity and overactivation of calpain-2, alleviating photoreceptor degeneration caused by Ca^2+^ imbalance. In summary, our data support the concept of a PARP–sirtuin-1–PGC-1α–PPAR–PARP feedback control that connects defective energy metabolism to photoreceptor degeneration. Specifically, our findings suggest that PPARα, PPARγ, and PGC-1α cooperate to preserve photoreceptor viability, highlighting PPAR-signaling as a promising target for future therapeutic interventions.

## Introduction

Retinitis pigmentosa (RP) is an inherited retinal degeneration (IRD) that affects approximately 1:4.000 people, with more than 2 million patients worldwide [1]. Causative genetic mutations initially cause rod dysfunction and degeneration, typically resulting in night blindness, followed by secondary cone degeneration. Most forms begin in the mid-peripheral retina and spread centrally and peripherally, causing progressive visual-field constriction, loss of central vision, and eventually legal blindness [2].

The mechanisms of RP photoreceptor degeneration remain only incompletely understood. While it was originally assumed that apoptosis was the primary mechanism of cell death [3], emerging evidence highlights the role of alternative pathways. For instance, cyclic guanosine monophosphate (cGMP)-mediated Ca^2+^ overload and over-activation of Ca^2+^-dependent calpain-type proteases have been suggested to cause photoreceptor cell death [4, 5]. Experimental evidence also points to an involvement of poly (ADP-ribose) polymerase (PARP) and excessive formation of poly (ADP-ribose) (PAR) polymers. Since PARP consumes high levels of nicotinamide dinucleotide (NAD^+^), its overactivation has been linked to dysfunction of other NAD^+^-dependent enzymes, notably of sirtuin-type histone deacetylases critical for mitochondrial homeostasis [6].

Photoreceptors require substantial energy to maintain their function and structural integrity [7]. For instance, a functional lipid metabolism is required to support photoreceptor function through membrane renewal, energy production, and cellular signaling [8–10]. Recent studies have highlighted links between RP and energy metabolism, suggesting that metabolic imbalance likely plays an important role in its pathogenesis [11, 12].

Peroxisome proliferator-activated factors (PPARs) are ligand-activated transcription factors and key regulators of cellular metabolism [13]. The PPAR family comprises PPARα, PPARγ, and PPARβ/δ, which regulate distinct but overlapping metabolic processes, particularly lipid metabolism [14]. PPARα regulates fatty acid β-oxidation (FAO), helping to maintain energy homeostasis [15]. PPARγ and PPARβ/δ participate in lipid metabolic regulation [16, 17]. Within the eye, PPARs have been related to pathologic processes in various retinal diseases, such as age-related macular degeneration and diabetic retinopathy [18–22].

PPAR transcriptional activity is modulated by co-regulators such as peroxisome proliferator-activated factor gamma coactivator 1-alpha (PGC-1α), a central regulator of cellular energy metabolism and mitochondrial function. PGC-1α is encoded by the *PPARGC1A* gene and forms complexes with PPARs to recruit additional cofactors, significantly enhancing their transcriptional activity on downstream target genes [23]. A key upstream regulator of PGC-1α is sirtuin-1, which can enhance PGC-1α activity through deacetylation, but its function may be compromised when excessive PARP activation depletes NAD^+^.

To investigate the roles of PPARs and PGC-1α in IRD, we analyzed gene expression profiles and utilized organotypic retinal explant cultures derived from the retinal degeneration 1 (*rd1*) mouse. The *rd1* mouse carries a mutation in the phosphodiesterase 6β gene, leading to the accumulation of cyclic guanosine monophosphate (cGMP) and progressive retinal degeneration [24]. Characterized by rapid rod photoreceptor loss, this spontaneous model has been extensively used in retinal degeneration research. Explants were treated with either subtype-specific PPAR agonists, PGC-1α activator, or PGC-1α inhibitor. We found that stimulation of PPAR or PGC-1α can prevent *rd1* photoreceptor degeneration. This protective PGC-1α/PPAR signaling appears to be dependent on the activity of sirtuin-type deacetylases and can be counteracted by excessive PARP activity. Our data stresses the importance of PGC-1α/PPAR signaling for photoreceptor survival and highlights the corresponding up- and down-stream pathways as targets for therapeutic intervention.

## Results

### Expression patterns of PPAR and PGC-1α in the retina

**Fig. 1:**
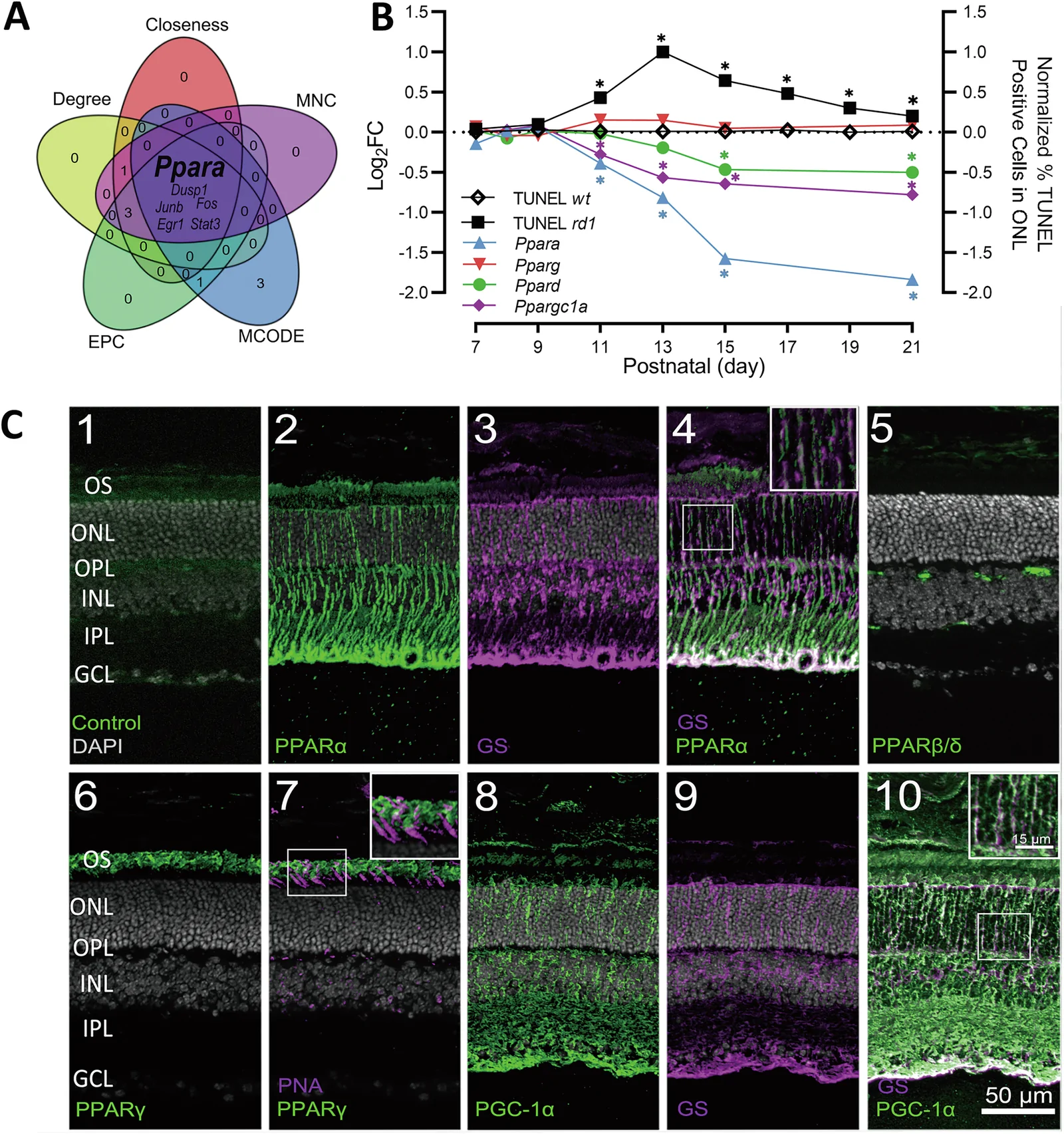
Analysis of PPAR and PGC-1α gene expression during *rd1* photoreceptor degeneration. **A** cytoHubba analysis of *rd1* transcriptomes using Degree, Closeness, Maximum Neighborhood Component (MNC), Edge Percolated Component (EPC), and MCODE algorithms identified *Ppara* as a shared hub gene. **B** Relative expression of *Ppara*, *g*, *d*, and *Ppargc1a* genes (Log_2_FC; left *y*-axis) during the first 3 postnatal weeks. Retinal cell death expressed as normalized percentage of TUNEL positive cells in the outer nuclear layer (ONL) is shown for comparison (right *y*-axis). **C** Immunostaining for PPARα, γ, β/δ and PGC-1α (green) at post-natal day (P) 30 in wild-type (*wt*) retina. DAPI (grey) was used as nuclear counterstain; negative control for secondary antibody shown in 1. Glutamine synthetase (GS; magenta) was used as Müller cell marker in 3.4 and 9.10; peanut agglutinin (PNA; magenta) was used to label cones in 7. Co-staining for PNA and PPARγ shows that PPARγ is expressed in rod outer segments (OS), but not in cones. Significance level: * = *p* < 0.05. OPL = outer plexiform layer, INL = inner nuclear layer, IPL = inner plexiform layer, GCL = ganglion cell layer; scale bar = 50 µm.

An initial study of the GSE62020 *rd1* transcriptome dataset [25], using MCODE cluster analysis [26] and cytoHubba [27] with the topological algorithms Degree, Closeness, Maximum Neighborhood Component (MNC), Edge Percolated Component (EPC) identified *Ppara* as a shared hub gene (Fig. 1A). Remarkably, another hub gene found in this process was early growth response protein-1 (EGR1), which had already been identified in a recent single-cell RNA sequencing study performed on *rd1* and wild-type (*wt*) retina [28]. This provided for an independent validation of the hub gene identification and motivated us to further investigate the role of PPARα in the progression of *rd1* retinal cell death. We then analyzed the same dataset, comparing *wt* and *rd1* retina at different post-natal (P) days. This transcriptomic data were related to the percentage of dying cells in ONL, as evidenced by the TUNEL assay, with *wt* dataset as baseline for the comparative analysis. In *rd1* retinas, retinal cell death increased significantly from P9 to P13, and was still significantly elevated at P21 (Fig. 1B). The expression of the *Ppara* gene encoding PPARα was markedly reduced at P11, *i.e*. at the onset of photoreceptor degeneration. Further analysis of other PPAR family members found a significant downregulation of *Ppard* (PPARβ/δ) expression only at P15, *i.e*. after the peak of ONL cell death. In contrast, *Pparg* (PPARγ) expression remained stable during the period of major photoreceptor loss up until P21 (Fig. 1B).

Since mRNA expression does not necessarily represent protein levels, we used immunofluorescence on mature P30 *wt* retina to determine PPAR protein expression and localization. PPARα was strongly expressed across the retina, spanning from the photoreceptor outer segments (OS) all the way through to the ganglion cell layer (GCL) (Fig. 1C2). To assess whether PPARα was expressed in Müller cells, we performed co-immunostaining with the Müller cell marker glutamine synthetase (GS). In the outer nuclear layer (ONL), PPARα signal appeared in fibers adjacent to GS label. In the GCL, PPARα appeared to be expressed in Müller cell endfeet (Fig. 1C4). PPARβ/δ signals were detected at the level of the outer plexiform layer (OPL) and at the inner border of the inner nuclear layer (INL), indicating an expression in inner retinal blood vessels (Fig. 1C5). PPARγ was mainly expressed in the OS of photoreceptors (Fig. 1C6). Co-staining of PPARγ with the cone marker peanut agglutinin (PNA) showed the PPARγ signal was localized outside of the PNA-positive regions, indicating predominant expression in rod photoreceptors (Fig. 1C7).

To exert their functions, PPARs require transcriptional co-regulators such as PGC-1α, which strongly enhances PPAR-mediated transcriptional activity on target genes [23]. Expression of *Ppargc1a* mRNA continuously declined concomitant with *rd1* photoreceptor loss (Fig. 1A), suggesting that its deficiency may contribute to cell death. Immunostaining showed strong PGC-1α expression in the INL, inner plexiform layer (IPL), GCL, and photoreceptor layer (Figure 1C8). PGC-1α immunofluorescence was closely related and partially overlapped with the Müller cell marker GS in the ONL, with clear co-localization with Müller cell endfeet within the GCL (Fig. 1C10).

### Effect of PPARs and PGC-1α on *rd1* photoreceptor death

**Fig. 2:**
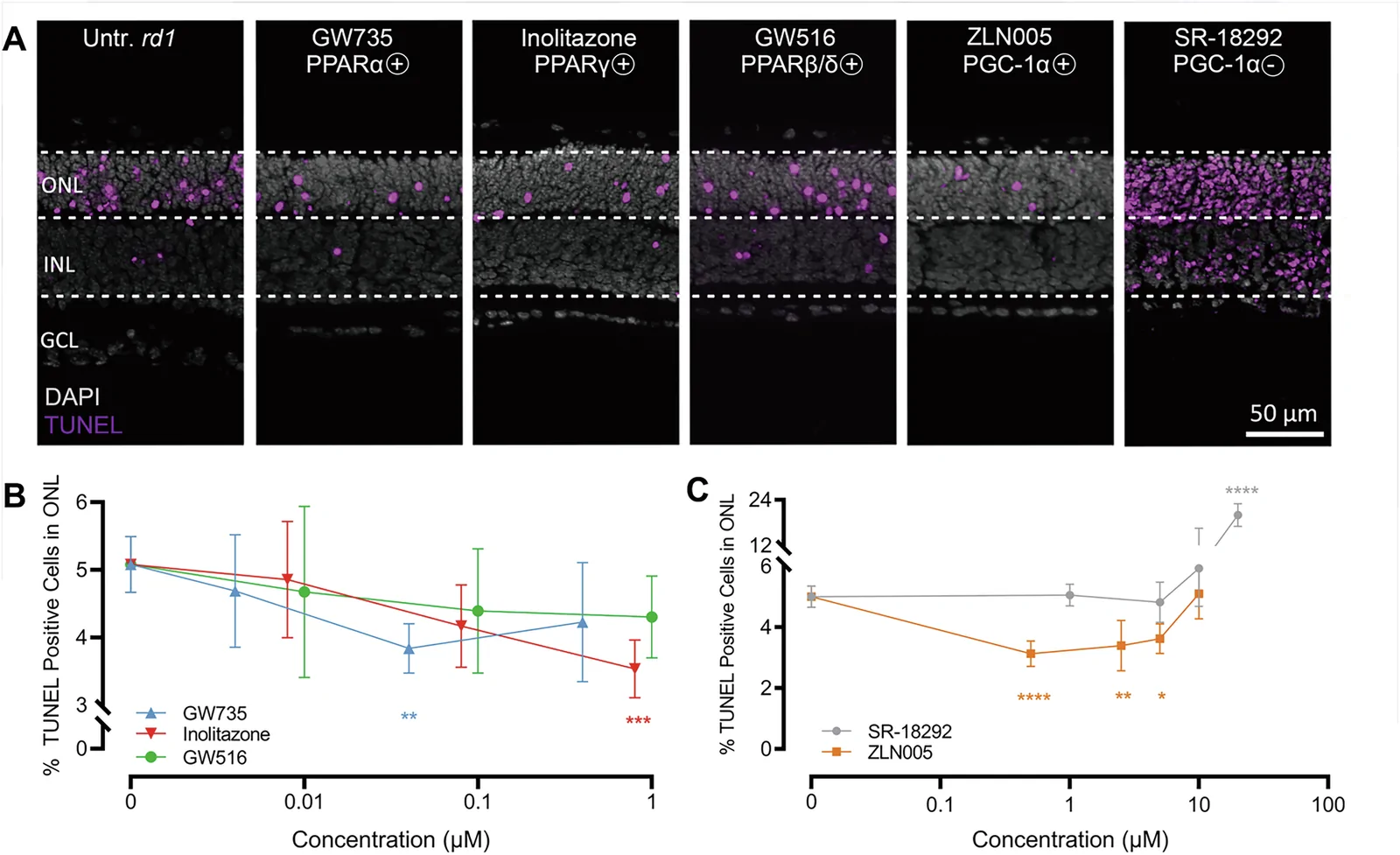
Photoreceptor cell death is decreased by PPAR and PGC-1α agonists. **A** TUNEL assay labeling dying cells (magenta) in *rd1* mouse retinal explant cultures. DAPI (grey) was used as nuclear counterstain. Untreated (Untr.) *rd1* retina (*n* = 16) was compared to treatment with either 0.04 µM PPARα agonist GW735 (*n* = 9), 0.8 µM Inolitazone (PPARγ agonist) (*n* = 12), 1 µM GW516 (PPARβ/δ agonist) (*n* = 10), 0.5 µM ZLN005 (PGC-1α activator) (*n* = 10), or 20 µM SR-18292 (PGC-1α inhibitor) (*n* = 6). **B** Dose-response curves for GW735, Inolitazone, and GW516 in *rd1* retinal cultures. In the outer nuclear layer (ONL), 0.04 µM GW735 and 0.8 µM Inolitazone, respectively, significantly reduced cell death. **C** Dose-response curves for ZLN005 and SR-18292. ZLN005, at 0.5–5 µM, significantly reduced ONL cell death, while 20 µM SR-18292 increased ONL cell death. Statistical testing: One-way ANOVA and Dunnett’s multiple comparisons test. Error bars represent SD; significance levels: ns = *p* > 0.05; * = *p* < 0.05; ** = *p*  <  0.01; *** = *p*  <  0.001; **** = *p* < 0.0001; INL = inner nuclear layer, GCL = ganglion cell layer; scale bar = 50 µm.

To explore potential links between PPAR activity and photoreceptor degeneration, we treated *rd1* and *wt* organotypic retinal explants with the PPARα agonist GW590735 (abbreviated as GW735), the PPARγ agonist Inolitazone dihydrochloride (hereinafter called Inolitazone), and the PPARβ/δ agonist GW501516 (abbreviated as GW516). The effects of these treatments were assessed using the TUNEL assay to quantify cell death in the ONL, and dose-response curves were established for each agonist. In *wt* retinal explants, the number of TUNEL-positive cells in the ONL was low (Fig. S1A, B and Table S1B). In untreated *rd1* explants, ONL cell death was significantly increased (Fig. 2A, B and Table S1A). Treatment with GW735 and Inolitazone markedly reduced photoreceptor cell death in *rd1* retina, and effective concentrations were well tolerated in *wt* retinas (Figs. 2A, B; S1A, B; and Table S1A, B). However, treatment with GW516 did not lead to a significant reduction of TUNEL-positive cells in the *rd1* ONL, yet, at the same concentration, it significantly increased *wt* retina ONL cell death (Figs. 2A, B; S1A, B; and Table S1A, B). Taken together, treatment with PPARα and PPARγ agonists appeared to significantly delay *rd1* photoreceptor degeneration.

To functionally characterize the role of the PPAR coactivator PGC-1α, we applied the PGC-1α activator ZLN005 and the PGC-1α inhibitor SR-18292, respectively, to retinal *rd1* and *wt* retinal explant cultures. In *rd1* explants, SR-18292 significantly increased the number of TUNEL-positive cells in the ONL to 19.79% (±2.16 SD, *n* = 6) (Fig. 2A, C and Table S1A), whereas in *wt* retinas, it caused only 2.35% (±0.60 SD, *n* = 5) cell death (Fig. S1A, B and Table S1B). This indicates that the effect of SR-18292 was not due to general cytotoxicity but instead was mediated through a specific mechanism in *rd1* retinas. Conversely, ZLN005 significantly reduced photoreceptor cell death in *rd1* retinas already at relatively low concentrations (Fig. 2A, C and Table S1A).

### ZLN005 treatment enhances PGC-1α, PPARα, and PPARγ expression

RNA-seq showed significant declines in *Ppargc1a* and *Ppara* expression (*cf*. Figure 1B), while PGC-1α and PPARα were detected in the ONL and PPARγ in the OS (*cf*. Figure 1C), suggesting their potential functional interactions in photoreceptors. To investigate PGC-1α-PPAR interactions in photoreceptor neuroprotection, we assessed PPARα and PPARγ expression in ZLN005-treated *rd1* retinal explants.

**Fig. 3:**
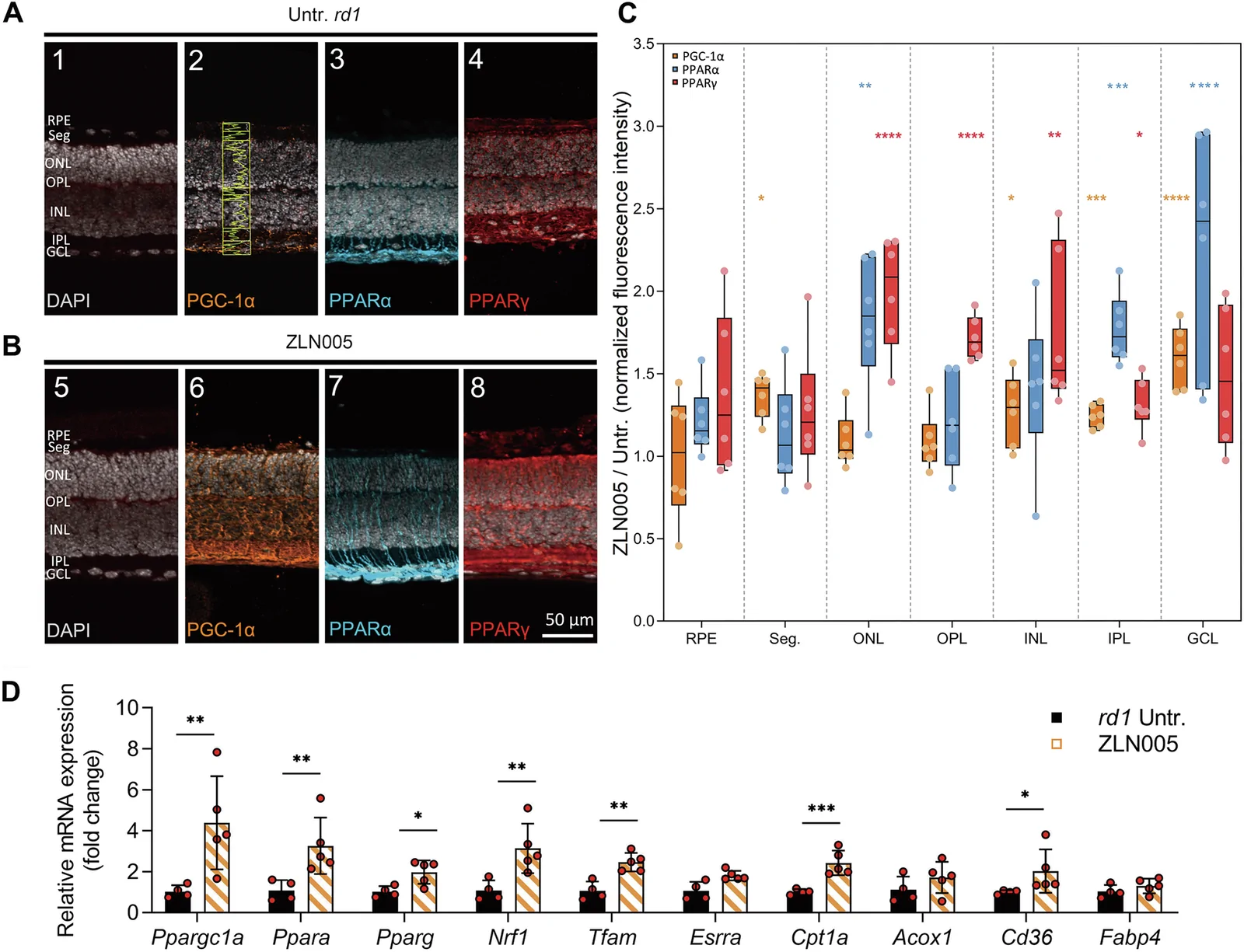
ZLN005 increases the expression of PGC-1α, PPARα, and PPARγ. **A** Immunostaining for PGC-1α (orange), PPARα (cyan), and PPARγ (red) in untreated (Untr.) *rd1* retina at post-natal day (P) 11. DAPI (grey) was used as nuclear counterstain. A2 shows example fluorescence intensity plot used for quantifications in (**C**). **B** Immunostaining for PGC-1α, PPARα, and PPARγ in P11 *rd1* retina treated with PGC-1α activator ZLN005. **C** Box and whisker plot for fluorescence intensity levels of PGC-1α, PPARα, and PPARγ in retina treated with the PGC-1α activator ZLN005 (*n* = 6). Data normalized relative to untreated control for each retinal layer. **D** RT-qPCR was performed to quantify mRNA levels of *Ppargc1a*, *Ppara*, *Pparg, Nrf1*, *Tfam*, *Esrra*, *Cpt1a*, *Acox1*, *Cd36*, and *Fabp4* in *rd1* untreated (*n* = 4) and ZLN005 treated (*n* = 5) retinal explant cultures. Statistical testing: Student’s *t*-test. Error bars represent SD; significance levels: ns = *p* > 0.05; * = *p* < 0.05; ** = *p* < 0.01; *** = *p* < 0.001; **** = *p* < 0.0001. RPE = retinal pigment epithelium; Seg. = photoreceptor inner and outer segments; ONL = outer nuclear layer; OPL = outer plexiform layer; INL = inner nuclear layer; IPL = inner plexiform layer; GCL = ganglion cell layer; scale bar = 50 µm.

The PGC-1α expression pattern found in in vitro retinal explant cultures was similar to that seen in the in vivo retina (compare Fig. 1C with Fig. 3A). Quantitative immunofluorescence analysis showed that in ZLN005-treated *rd1* retinal explants, the fluorescence intensity of PGC-1α in the photoreceptor segments, INL, IPL, and GCL was significantly stronger than that in the untreated *rd1* explants (Fig. 3A–C). Following ZLN005 treatment, PPARα fluorescence intensity was also significantly increased in the ONL, IPL, and GCL (Fig. 3A–C); PPARγ signals increased in the ONL, OPL, INL, and IPL (Fig. 3A–C).

Consistent with these observations, RT-qPCR demonstrated significantly increased mRNA expression of *Ppargc1a*, *Ppara*, and *Pparg* following ZLN005 treatment (Fig. 3D). PGC-1α is associated with mitochondrial transcriptional regulation through nuclear respiratory factor 1 (NRF1), estrogen-related receptor α (ERRα, encoded by *Esrra*), and mitochondrial transcription factor A (TFAM) [29, 30]. RT-qPCR analysis showed that ZLN005 significantly increased *Nrf1* and *Tfam* mRNA levels. PGC-1α is also involved in lipid metabolic regulation through PPARα and PPARγ [31]. As PPARα is closely associated with FAO, we examined the expression of carnitine palmitoyltransferase 1A (CPT1A) and acyl-CoA oxidase 1 (ACOX1), which are involved in mitochondrial and peroxisomal FAO [32, 33], respectively. ZLN005 treatment significantly increased *Cpt1a* but not *Acox1* mRNA expression. In parallel, two PPARγ-related genes, fatty acid-binding protein 4 (FABP4) and the fatty acid translocase (CD36/FAT), involved in fatty acid transport [31, 34], were analyzed. Our data showed that mRNA level of *Cd36* was significantly increased.

Taken together, our data indicated that ZLN005 treatment significantly enhanced the expression of PGC-1α, PPARα, and PPARγ, as well as a number of genes downstream of PGC-1α/PPAR signaling, which overall supported *rd1* photoreceptor viability.

### Modulation of PGC-1α and PPARs limits PARP activity in *rd1* photoreceptors

PGC-1α activity is tightly controlled via sirtuin-1-mediated deacetylation, and we therefore investigated if selective activation of sirtuin-1 using resveratrol could restore its protective function [35]. However, in retinal explant cultures, resveratrol treatment alone was insufficient to mitigate photoreceptor degeneration (Fig. S3A). This lack of effect might have been due to the circumstance that NAD^+^, *i.e.*, the substrate for sirtuin activity, was depleted by (excessive) PARP activity [36]. We therefore utilized the specific inhibitor PJ34 to suppress PARP activity and to reduce NAD^+^ consumption. In line with previous data [37], PJ34 treatment alone already significantly reduced photoreceptor cell death (Fig. S3B). The combination of sirtuin-1 activation with resveratrol and PARP inhibition with PJ34 led to a further, statistically significant decrease in the number of ONL TUNEL-positive cells (Fig. S3B). These data suggested that PARP, possibly via its use of NAD^+^, had an important effect on PPAR activity.

**Fig. 4:**
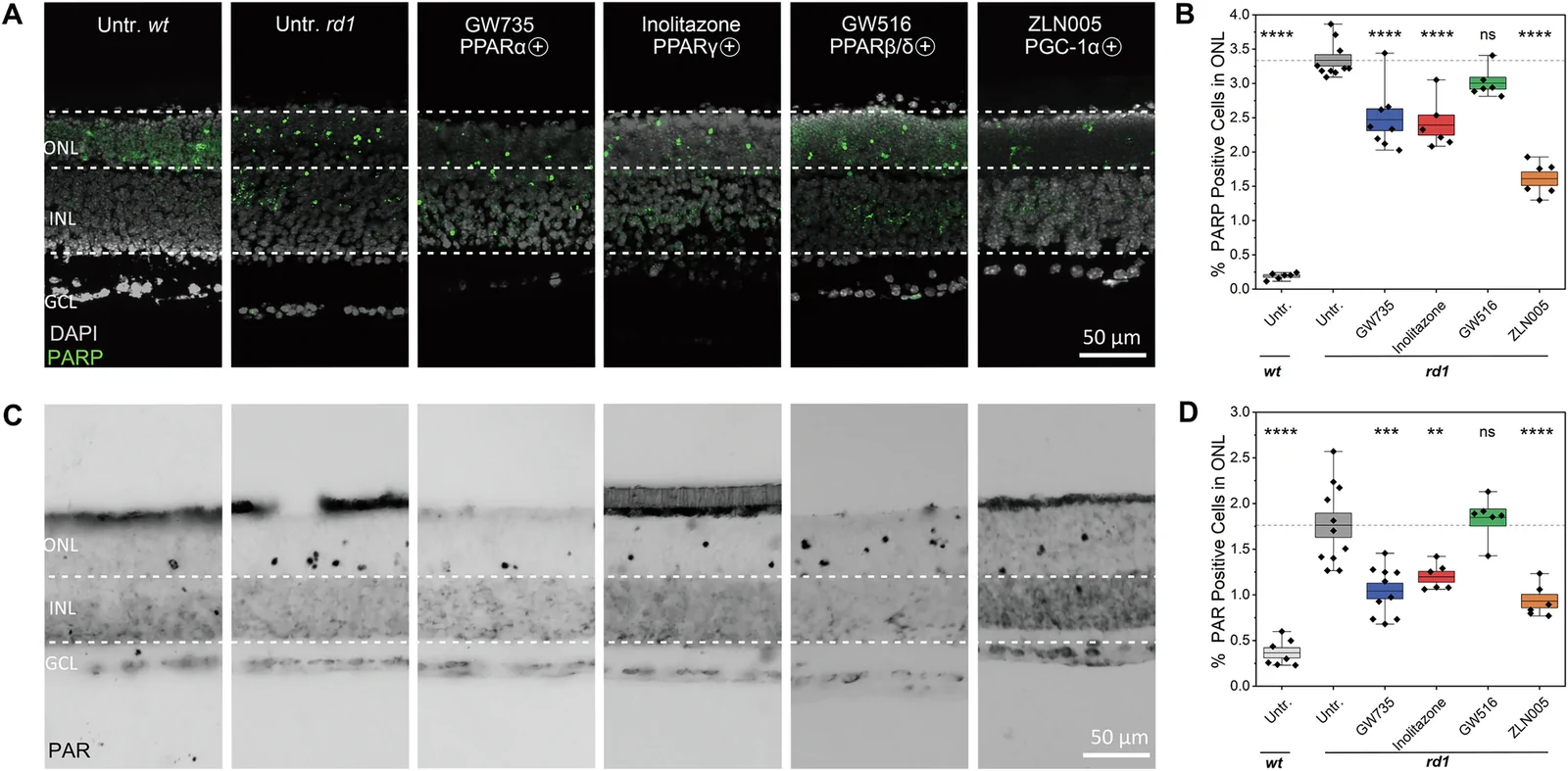
GW735, inolitazone, and ZLN005 affect PARP activity and PAR generation. **A** PARP activity assay (green) was performed in *rd1* and wild-type (*wt*) retinal explant cultures, with DAPI (grey) as nuclear counterstain. Untreated (Untr.) *wt* (*n* = 6) and *rd1* retina (*n* = 10) were compared to *rd1* retina treated with PPARα agonist GW735 (*n* = 8), PPARγ agonist Inolitazone (*n* = 6), PPARβ/δ agonist GW516 (*n* = 6), and PGC-1α activator ZLN005 (*n* = 6). **B** Box-and-whisker plots showing the percentage of PARP activity-positive cells in the outer nuclear layer (ONL). The dashed line indicates *rd1* Untr. situation, data points below this threshold indicate protective effects, data points above suggest destructive effects. **C** PAR staining (black) was performed in *wt* and *rd1* retinal explant cultures. *n* = 7, 11, 10, 6, 6, and 6 for untreated *wt*, untreated *rd1*, GW735, Inolitazone, GW516, and ZLN005 groups, respectively. **D** Untreated *wt* and *rd1* retina were compared to drug-treated retina as in B. Statistical testing: Two-way ANOVA and Dunnett’s multiple comparisons test. Error bars represent SD; significance levels: ns = *p* > 0.05; * = *p* < 0.05; ** = *p* < 0.01; *** = *p* < 0.001; **** = *p* < 0.0001. INL = inner nuclear layer, GCL = ganglion cell layer; scale bar = 50 µm.

To further explore the link between PGC-1α/PPAR signaling and PARP activity, in situ PARP activity assays and immunostaining for PAR were performed (Figs. 4A–D; S2A–D; and Tables S2A, B; S5A–D). PARP activity and PAR-positive cells were lower in *wt* retinas compared with *rd1*, as shown previously [37]. However, ZLN005 treatment significantly reduced PARP activity and PAR generation in *rd1* ONL. The utilization of GW735 and Inolitazone also reduced PARP and PAR accumulation. In contrast, activation of PPARβ/δ using GW516 neither reduced PARP activity nor did it suppress PAR production. These results indicate that PGC-1α, PPARα, and PPARγ activation contribute to photoreceptor survival, at least in part, by suppressing PARP activity and its downstream effects. Taken together, these data suggest the existence of a feedback loop between the activities of PARP, sirtuin, PPAR, and PGC-1 in *rd1* photoreceptors.

### ZLN005 and Inolitazone regulate calpain activity

**Fig. 5:**
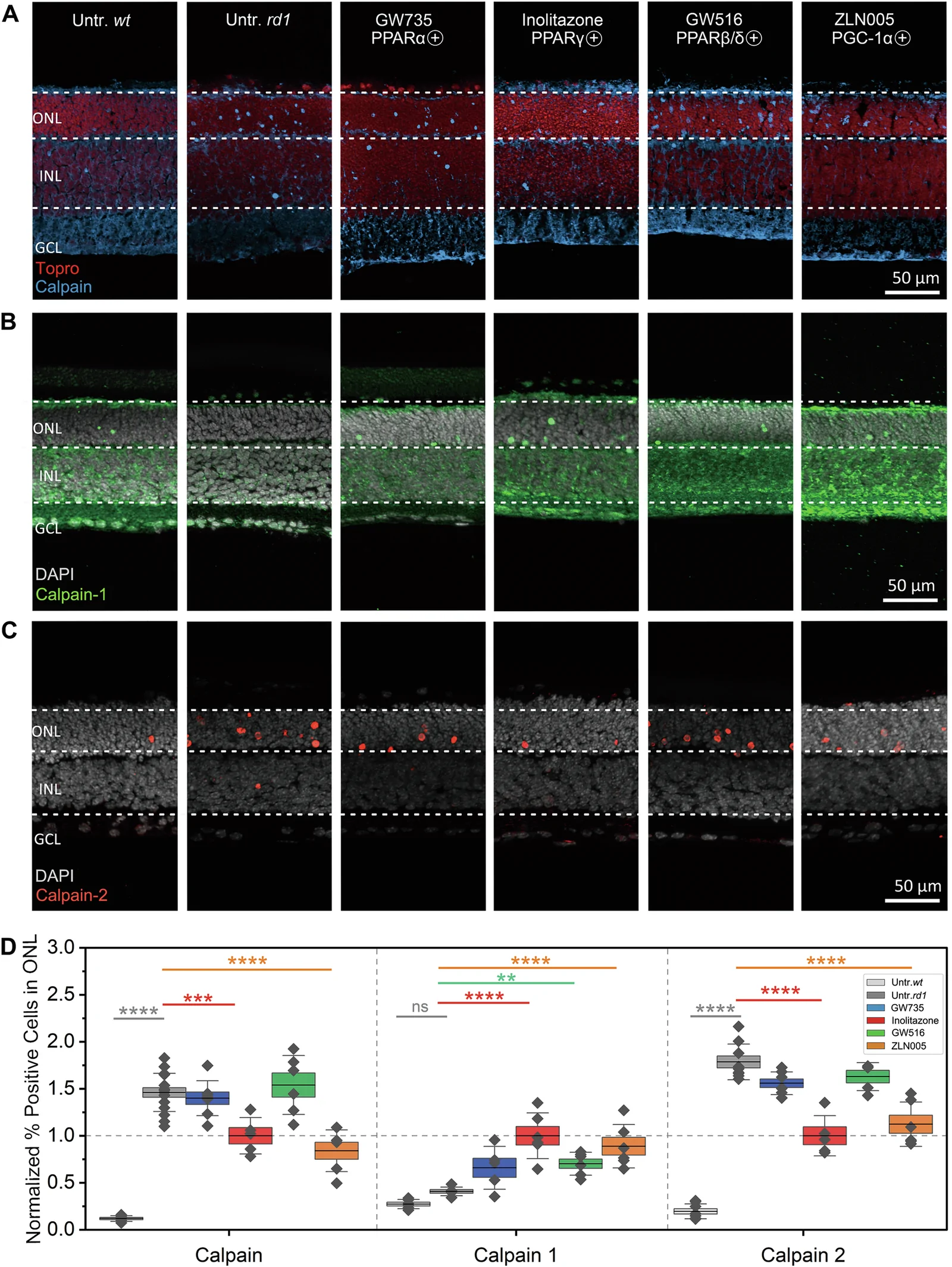
PPARs and PGC-1α differentially regulate calpain activity. **A** Calpain activity assay (blue) in wild-type (*wt*) and *rd1* retinal explant cultures, nuclear counterstain with ToPro3 (red). Untreated (Untr.) *wt* (*n* = 7) and *rd1* retina (*n* = 16) were compared to *rd1* retina treated with PPARα agonist GW735 (*n* = 6), PPARγ agonist Inolitazone (*n* = 5), PPARβ/δ agonist GW516 (*n* = 6), and PGC-1α agonist ZLN005 (*n* = 6). **B** Activated calpain-1 immunostaining (green) in *wt* and *rd1* retinal explant cultures with DAPI (grey) as nuclear counterstain. *n* = 6, 10, 6, 6, 6, and 6 for untreated *wt*, untreated *rd1*, GW735, Inolitazone, GW516, and ZLN005 groups, respectively. **C** Activated calpain-2 immunostaining (red) in *wt* and *rd1* retinal explant cultures with DAPI (grey) as nuclear counterstain. *n* = 8, 9, 7, 5, 5, and 6 for untreated *wt*, untreated *rd1*, GW735, Inolitazone, GW516, and ZLN005 groups, respectively. **D** Box-and-whisker plot showing normalized percent calpain activity-positive cells and displaying calpain-1 and -2 activation in outer nuclear layer (ONL). Data was normalized to Inolitazone treatment (grey dashed line), which showed the strongest effect on calpain activity, according to the formula: ${{{\rm{X}}}}_{{{\rm{scaled}}}}={{{\rm{X}}}}/{\bar{{{\rm{X}}}}}_{{{\rm{Inolitazone}}}}$, here, χ is the positive cells for the indicated condition, and ${\bar{\chi}}_{{{\rm{Inolitazone}}}}$, is the mean of Inolitazone treatment. Statistical comparisons were then performed using untreated *rd1* as control to determine significant differences among treatments. Statistical testing: Two-way ANOVA and Dunnett’s multiple comparisons test. Error bars represent SD; significance levels: ns= *p* > 0.05; * = *p* < 0.05; ** = *p* < 0.01; *** = *p* < 0.001; **** = *p* < 0.0001. INL = inner nuclear layer, GCL = ganglion cell layer; scale bar = 50 µm.

Photoreceptor degeneration is also associated with calpain-mediated proteolysis and cellular damage [4, 38]. To investigate calpain activity, we first employed a general in situ calpain activity assay. While calpain activity was relatively low in *wt* compared to the *rd1* situation, treatment with Inolitazone and ZLN005 significantly reduced, while GW735 and GW516 did not affect, overall calpain activity (Figs. 5A, D; S2A–D; and Tables S3A; S5A–D).

The two major calpain isoforms involved in neurodegenerative processes are calpain-1 and calpain-2, where calpain-1 activity is seen as mostly neuroprotective while activity of calpain-2 is likely destructive [39]. To study these two isoforms, we used immunolabelling for activated calpain-1 and calpain-2 and quantified the numbers of positive cells in the ONL (Figs. 5B–D; S2; and Table S3B, C; S5A–D). The extent of calpain-1 activation was similar between *rd1* and *wt* retinas. Calpain-2 activation, however, was high in *rd1* retina when compared with *wt* retina. Inolitazone and ZLN005 significantly increased calpain-1 activation, while reducing calpain-2 activation. PPARβ/δ activation only increased the number of calpain-1-positive cells in the *rd1* ONL without inhibiting calpain-2 activation. There was no significant effect on the fraction of activated calpain-1 or calpain-2-positive cells in the *rd1* ONL after treatment with GW735.

## Discussion

**Fig. 6:**
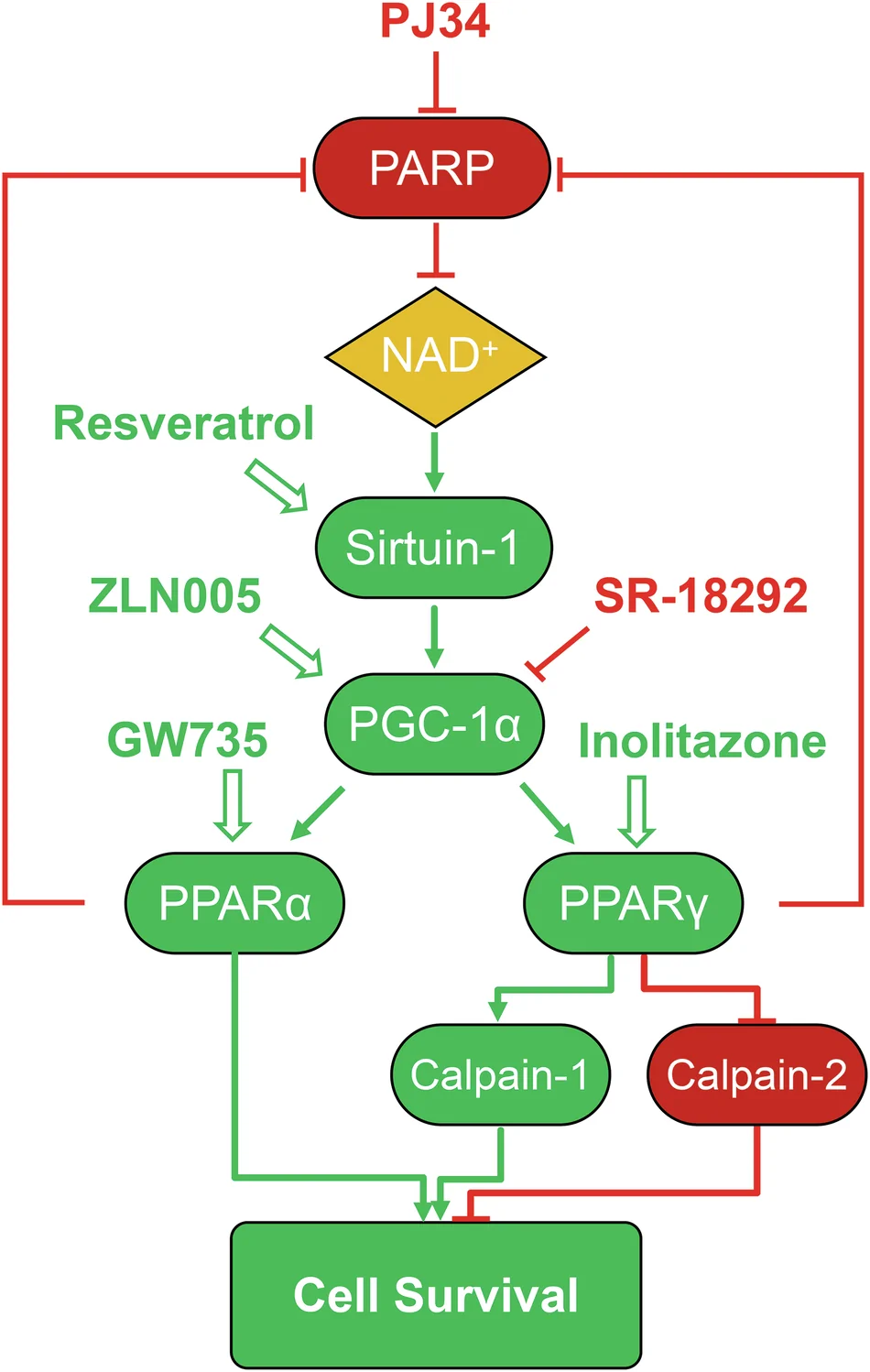
PGC-1α and PPAR retinal pro-survival signaling and experimental interventions. The PARP enzyme consumes NAD^+^, a substrate required for the function of sirtuin-1. Sirtuin-1 mediated deacetylation activates PGC-1α, which in turn functions as a coactivator for the nuclear receptors PPARα and PPARγ. This synergistic interaction drives transcriptional programs that support cell survival. While PPARα promotes survival independent of calpain activity, PPARγ signaling involves increased activation of the protective calpain-1 and decreased activation of the destructive calpain-2 isoform. This signaling network can be modulated by a variety of specific inhibitors and activators, potentially improving cell survival. While PARP can be inhibited by PJ34, resveratrol enhances sirtuin-1 activity as long as sufficient NAD^+^ is available. ZLN005 is described as a direct activator of PGC-1α, leading to its upregulation and activation. Conversely, SR-18292 inhibits PGC-1α. PPARα and PPARγ can also be directly targeted by the specific pharmacological agonists GW735 and Inolitazone, respectively. Red color indicates inhibition/downregulation, while green indicates activation/upregulation.

In the central nervous system, PPARs play an important role in regulating cellular metabolism, as well as cell death and survival. Here, we show that PGC-1α and PPAR signaling cooperate to maintain retinal homeostasis and photoreceptor cell survival in the context of IRD (Fig. 6). The protective effects associated with PGC-1α and PPAR signaling are likely subject to positive regulation from sirtuin activity, while PARP activity may provide negative feedback. Moreover, the excessive activation of calpain-type proteases, notably calpain-2, appears to be situated down-stream of PGC-1α and PPAR-signaling.

### PPARα-, PPARγ-, and PGC-1α activities are associated with photoreceptor survival

Pharmacological targeting of PPARα and PPARγ showed protective effects on *rd1* photoreceptors. A decline of PPARα expression was associated with photoreceptor degeneration, while its selective agonist GW735 reduced cell death. Although PPARγ gene expression remained largely unchanged during the initial stages of retinal degeneration, its agonist Inolitazone significantly enhanced photoreceptor survival. In contrast, PPARβ/δ was downregulated only at later stages of *rd1* rod degeneration, suggesting that this change reflects disease progression rather than being a primary driver of cell death. Consistent with this idea, the PPARβ/δ agonist GW516 did not alleviate photoreceptor loss in *rd1* retina. This concept is in accordance with previous work indicating widespread retinal expression of PPARβ/δ but only a minor role in maintaining retinal homeostasis [40]. Therefore, unlike PPARα and PPARγ, targeting PPARβ/δ appears to have limited therapeutic potential for use in RP patients.

A general target of PPAR signaling is lipid metabolism [41]; in particular, PPARα was found to be important for retinal FAO and photoreceptor survival [32]. The loss of PPARγ impairs fatty acid (FA) metabolism and leads to retinal abnormalities [32, 42]. These findings raise the possibility that the effects of PPARα or PPARγ on photoreceptor survival in RP may be linked to their roles in lipid metabolism.

PGC-1α is also recognized to play a key role in regulating lipid metabolism [43], and its loss in retinal pigment epithelium downregulates PPARs expression, accompanied by reduced FAO, impaired FA transport, and increased lipid peroxidation [31]. In our study, ZLN005 enhanced photoreceptor survival and increased *Ppara* and *Pparg* expression, which was accompanied by increased expression of *Cpt1a*, a downstream target gene of PPARα involved in FAO [32], and *Cd36*, a PPARγ-related gene associated with FA transport [34]. Overall, these findings point to a potential association between PGC-1α/PPAR signaling and lipid homeostasis. Based on our findings and previous studies [30], photoreceptor protection associated with PGC-1α/PPAR signaling may be related to mitochondrial regulation and biogenesis [44, 45].

### Bidirectional control of PPAR-signaling via PARP activity

Full activation of PGC-1α is achieved by sirtuin-1-mediated deacetylation [46], and thus the sirtuin activator resveratrol may exert a neuroprotective function [35, 47]. Yet, in *rd1* mice, resveratrol failed to prevent photoreceptor degeneration, consistent with a previous study on N-methyl-N-nitrosourea (MNU) -induced rapid photoreceptor degeneration, in which resveratrol also did not prevent the decline of retinal function [48]. To be active, sirtuin-1 requires NAD^+^ as substrate, which, on the other hand, is also the substrate for PARP activity. The PARP overactivation observed in *rd1* photoreceptors likely depletes NAD^+^ [4, 49] and limits sirtuin-1 activity. This interdependence would be consistent with previous studies [50] and is supported by the results obtained from the combined treatment using resveratrol to activate sirtuin-1 and PJ34 to block PARP, which significantly decreased *rd1* ONL cell death. Taken together, our data point to a role of PARP as an upstream regulator of PGC-1α/PPARs -signaling.

On the other hand, treatment with PGC-1α, PPARα, and PPARγ activators reduced photoreceptor PARP activity and PAR accumulation, indicating that PARP may also be a downstream effector. It has been reported that PGC-1α, PPARα, and PPARγ reduce superoxide production and activate the expression of antioxidant enzymes [51–55]. Thus, PGC-1α/PPAR-signaling may reduce PARP activity, possibly by maintaining redox homeostasis and limiting oxidative damage to DNA. Overall, our data support the concept of a PARP–sirtuin-1–PGC-1α–PPAR–PARP bidirectional feedback loop, linking photoreceptor degeneration to a deficient energy metabolism (Fig. 6).

### PPARγ and PGC-1α regulate calpain proteolytic activity

Regarding calpains, we found that the activation of PGC-1α and PPARγ produced a marked downregulation of overall calpain activity and specifically a reduction of calpain-2 activation. Since calpain-2 activation requires high intracellular Ca^2+^-levels, we hypothesize a role of PPARγ in the maintenance of cellular Ca^2+^ homeostasis. This is supported by the fact that PPARγ agonists are known to maintain mitochondrial Na^+^/Ca^2+^ exchanger levels, allowing mitochondria to buffer intracellular Ca^2+^ [56]. Furthermore, PGC-1α can prevent mitochondrial Ca^2+^ overload by regulating expression of the mitochondrial calcium uniporter complex [57, 58]. It is thus plausible that PGC-1α together with PPARγ may regulate intracellular Ca^2+^-levels and indirectly calpain activation. Eventually, this may lead to an excessive proteolysis, which, without intervention, would promote photoreceptor cell death. On the other hand, both PGC-1α and PPARγ activation resulted in increased activation of calpain-1, an isoform often associated with neuroprotection [39], confirming a recent study on the mechanisms of *rd1* photoreceptor degeneration [38].

In contrast, PPARβ/δ activation had no effect on photoreceptor calpain activity, a result that corresponds to its lack of effect on *rd1* ONL cell death, and possibly is explained by its localization to the retinal vasculature.

Perhaps the most interesting observation in this context is that PPARα activation, while reducing ONL cell death in the *rd1* model, did not significantly affect calpain activity. This indicates that the neuroprotection accomplished by PPARα is largely independent of Ca^2+^ homeostasis regulation. Altogether, these observations provide a rationale for a combined treatment of IRDs with PPARα and PPARγ agonists to exploit the full therapeutic power provided by different and independent neuroprotective mechanisms.

### Cooperation and differentiation of PPARα, PPARγ, and PGC-1α

The differential localization of PPARα, PPARγ, and PGC-1α suggests cell-specific roles in photoreceptor degeneration. PPARα and PPARγ were both augmented in the lipid membrane-rich photoreceptor OS, consistent with potential roles in lipid metabolism and OS structural maintenance [59]. Additionally, PPARα was widely expressed in the retina, and PGC-1α was distributed across the ONL, IPL, and INL, suggesting that their activities may extend beyond photoreceptors and involve multiple retinal cell types and layers, including Müller glia cells [60, 61]. It has been shown that the retina functions as an integrated metabolic system in which Müller cells support neuronal metabolism [7, 62]. Notably, although PGC-1α and PPARα showed limited colocalization with Müller cell markers in the ONL, both proteins were detected in Müller cell endfeet in the GCL. This localization suggests that PGC-1α/PPAR signaling may participate in Müller cell-mediated metabolic support and thereby indirectly influence photoreceptor energy homeostasis [7]. These findings suggest that the PGC-1α/PPAR pathway may act within a broader retinal metabolic network. In this context, PPARα, PPARγ, and PGC-1α may share some downstream mechanisms, including regulation of PARP activity [63, 64]. Nevertheless, the differential effects of PPARα and PPARγ agonists on calpain activity highlight that these pathways only partially overlap and may have distinct downstream effects in RP.

Given the rapid photoreceptor degeneration in *rd1* mice, future studies are planned to extend these findings to chronic RP models to evaluate the effects of PGC-1α-, PPARα-, and PPARγ-targeted interventions beyond the peak phase of rod loss, and to assess their potential to preserve cone survival and function. In addition, our findings on changes in the expression of genes associated with FA metabolism and mitochondrial homeostasis will need to be corroborated by future metabolomic, lipidomic, isotope-tracing, and mitochondrial functional analyses to determine whether these changes translate into functional metabolic remodeling in degenerating photoreceptors. Such studies may help to map out the larger PGC-1α/PPAR signaling network and to reveal a wider range of targets in IRDs beyond photoreceptors.

## Conclusion

The key finding of this work is that pharmacological targeting of PPARα, PPARγ, and PGC-1α can alleviate photoreceptor degeneration in *rd1* mouse retina. These factors may synergistically interact to improve photoreceptor viability through several potential mechanisms, including inhibition of calpain and PARP overactivation, as well as possible regulation of FAO and mitochondrial homeostasis. Moreover, their selective regulation reveals distinct neuroprotective mechanisms for different PPAR subtypes and PGC-1α, providing a foundation for future intervention strategies in degenerative retinal diseases that may be improved by addressing different targets in a combined approach.

## Materials and methods

### Animals

Wild-type (*wt*) C3H/HeA *Pde6b*^*+/+*^ and congenic C3H/HeA *Pde6b*^*rd1/rd1*^ mice (*rd1*) were used irrespective of sex. All efforts were made to minimize the number of animals used and their suffering. Mice were housed in the specified-pathogen-free (SPF) housing facility at the Tübingen Institute for Ophthalmic Research, under standard white cycling light, with free access to food and water.

### Analysis of RNA-seq data

The mRNA expression comparison between *wt* and *rd1* mouse employed the GSE62020 dataset downloaded from the Gene Expression Omnibus (GEO) database (https://www.ncbi.nlm.nih.gov/geo; information retrieved in July 2025). This dataset was previously reported [25]. To characterize *rd1* data, analysis was performed using R software (version 4.5.1) and the limma package (version 3.64.3) for normalization and differential expression analysis (fold change > 1.2, *p* < 0.05) [65].

### Organotypic retinal explant cultures

Retinas were used to generate explants following the standard protocol as previously described [66]. Mice were decapitated at postnatal day 5 (P5), the heads cleaned with 70% ethanol, and the eyes removed under aseptic conditions. The explants were cultured on a polycarbonate membrane (Order No.: 83.3930.040; 0.4 µm TC-inserts; Sarstedt, Nümbrecht, Germany) with complete R16 medium (Order No: 07491252A, Gibco, Paisley, UK) including supplements [66]. On the day of culture, retinal explants from left and right eyes of each mouse were randomly assigned to different experimental groups; subsequent processing was identical across groups. No blinding was used for sample allocation. The medium was exchanged every 2 days, with treatment added at P7 and P9. Cultures were treated with 0.04 µM GW590735 (HY-106278, MedChemExpress, Sollentuna, Sweden), 0.8 µM Inolitazone dihydrochloride (HY-14792B; MedChemExpress), 1 µM GW501516 (HY-10838; MedChemExpress), 0.5 µM ZLN005 (HY-17538; MedChemExpress), 20 µM SR-18292 (HY-101491; MedChemExpress), 20 µM resveratrol (501-36-0; Sigma, St. Louis, MO, USA), 6 µM PJ34 (Alexis Biochemicals, Lausen, Switzerland). All compounds were dissolved in DMSO at a final medium concentration of no more than 0.1% DMSO. Culturing was stopped at P11 either by fixation with 4% paraformaldehyde (PFA) or without fixation and direct freezing in liquid N_2_ for all treatment conditions. Explants were embedded in Tissue-Tek O.C.T. compound (Sakura Finetek Europe B.V., Alphen aan den Rijn, The Netherlands), then cut into 14 µm sections using a cryostat (CryoStar NX50 OVP; Thermo Fisher Scientific, Runcorn, UK), after which they were dried and stored at -20 °C for future use.

### TUNEL assay

A terminal deoxynucleotidyl transferase dUTP nick end labeling (TUNEL) assay kit (Roche Diagnostics, Mannheim, Germany) was used to label dying cells in retinal tissue sections. Fixed sections were rehydrated with 0.1 M phosphate-buffered saline (PBS) at room temperature (RT), while unfixed sections were first fixed in 4% PFA for 10 min, then washed with PBS. Subsequent steps were identical for both fixed and unfixed sections. Sections were incubated in Tris-buffered saline (TBS; 1 µL enzyme/mL) with proteinase K (1.5 µg/µL) at 37 °C for 5 min to inactivate nucleases, followed by treatment with 70:30 ethanol-acetic acid mixture at −20 °C for 5 min and three washes in TBS. Sections were then incubated with blocking solution (10% normal goat serum (NGS)), 1% bovine serum albumin (BSA), and 1% fish gelatin in PBS with 0.3% Triton X-100 for 1 h at RT, followed by overnight incubation with TUNEL staining solution at 4 °C. Finally, sections were washed three times with PBS (5 min each), mounted in Vectashield with DAPI (Vector Laboratories Inc., Burlingame, CA, USA), and imaged by microscopy.

### Calpain activity assay

Unfixed retinal tissue sections were rehydrated in calpain reaction buffer (CRB) (5.96 g HEPES, 4.85 g KCl, 0.47 g MgCl_2_, and 0.22 g CaCl_2_ in 100 mL ddH2O; pH 7.2) with 2 mM dithiothreitol (DTT) for 15 min. Then, sections were incubated with 25 µM tBOC-Leu-Met-CMAC (A6520; Thermo Fisher Scientific, OR, USA) for 3 h at 37 °C. This was followed by two washes with PBS and 12 min incubation with ToPro3 (1:1000 in PBS; T3605, Invitrogen, Carlsbad, USA). Afterwards, sections were mounted using Vectashield without DAPI (Vector Laboratories) for immediate microscopic visualization.

### PARP activity

The in situ PARP activity assay was performed as published previously [49]. In brief, unfixed retinal tissue sections were rehydrated in PBS for 10 min, then incubated at 37 °C for 3 h in a reaction mixture (10 mM MgCl_2_, 1 mM DTT, and 50 µM 6-Fluo-10-NAD^+^ (Cat. Nr.: N 023; Biolog, Bremen, Germany) in 100 mM Tris buffer with 0.2% Triton X100, pH 8.0). After three 5-min washes in PBS, sections were mounted in Vectashield with DAPI (Vector Laboratories).

### PAR-DAB staining

3,3′-diaminobenzidine (DAB) staining was performed on fixed sections to detect PAR generation. To quench endogenous peroxidase activity, sections were incubated in 40% Methanol and 10% H_2_O_2_ in PBS with 0.3% Triton X-100 (PBST) for 20 min. Sections were further incubated with 10% NGS in PBST for 30 min, followed by anti-PAR antibody (1:200; Enzo Life Sciences, Farmingdale, New-York) incubation overnight at 4 °C. After three 10 min washes in PBS, sections were incubated with biotinylated secondary antibody (1:150, Vector in 5% NGS in PBST) for 1 h. This was followed by staining with the Vector ABC Kit (Vector Laboratories, solution A and solution B in PBS, 1:150 each) for 1 h. DAB staining solution (0.05 mg/mL NH_4_Cl, 200 mg/mL glucose, 0.8 mg/mL nickel ammonium sulfate, 1 mg/mL DAB, and 0.1 vol. % glucose oxidase in phosphate buffer) was applied evenly, incubated for 2 min, and immediately rinsed with phosphate buffer to stop the reaction. Sections were mounted in Aquatex (Merck, Darmstadt, Germany).

### Immunohistochemistry

Fixed eyecups or retinal explant slides were rehydrated in PBS for 15 min at RT. Afterwards, sections were incubated with blocking solution (10% NGS, 1% BSA, 0.3% PBST) for 1 h. The primary antibodies (Table 1) were diluted in blocking solution and incubated overnight at 4 °C. Rinsing with PBS 3 times 10 min each was followed by incubation with secondary antibody (Table 1) for one hour. The sections were rinsed with PBS 3 times, 10 min each, and mounted with mounting medium with DAPI (Vector Laboratories).

**Table 1:** Primary and secondary antibodies used in the study, providers, and dilutions.

Antibody	Provider, Cat. No.	Dilution
Rabbit polyclonal anti-calpain-1	Abcam, ab39170	1:100
Rabbit polyclonal anti-calpain 2	Abcam, ab39165	1:200
Mouse monoclonal anti-glutamine synthetase (GS)	Abcam, MAB302	1:1000
Rabbit polyclonal anti-PPAR alpha	Novus, NB600-636	1:100
Rabbit polyclonal anti-PPAR gamma	Proteintech, 16643-1-AP	1:200
Mouse monoclonal anti-PPAR delta	Immunoway, YM3601	1:100
Mouse monoclonal anti-PGC-1 alpha	Proteintech, 66369-1-lg	1:200
Peanut agglutinin (PNA)	Vector Laboratories, FL-1071	1:500
Goat-anti-rabbit AlexaFluor568 (AF568)	Molecular Probes, A11036	1:350
Goat-anti-rabbit AlexaFluor488 (AF488)	Molecular Probes, A11034	1:350
Goat-anti-mouse AlexaFluor568 (AF568)	Molecular Probes, A11031	1:350

### Microscopy and image analysis

Images were captured using a Zeiss Imager Z.2 fluorescence microscope equipped with ApoTome2, an Axiocam 506 mono camera, and an HXP-120V fluorescent lamp (Carl Zeiss Microscopy, Oberkochen, Germany). The excitation (*λ*_Exc_)/emission (*λ*_Em_) characteristics of the filter sets used for the different fluorophores were as follows (in nm): DAPI (*λ*_Exc_ = 369 nm, *λ*_Em_ = 465 nm), AF488 (*λ*_Exc_ = 490 nm, *λ*_Em_ = 525 nm), AF568 (*λ*_Exc_ = 578 nm, *λ*_Em_ = 602 nm), and ToPro (*λ*_Exc_ = 642 nm, *λ*_Em_ = 661 nm). Images were captured at 20× magnification using the Z-stack mode of Zen 2.3 blue edition software. Sections of 14 μm thickness were analyzed using 15 Apotome Z-planes. Data were obtained from ex vivo retinas or explants derived from at least three animals for each condition.

For quantification, images were captured at six random locations per retinal section. The percentage of positive cells was determined as follows: The average area occupied by a photoreceptor cell (i.e., cell size) was determined by counting the number of DAPI-stained nuclei in six different rectangular areas of the retina. The total number of photoreceptor cells was estimated by dividing the given ONL area by this average cell size. The number of positively labeled cells (TUNEL, PARP, PAR, calpain, calpain-1, calpain-2) in the ONL was counted manually. The percentage of positive cells was then calculated by dividing the number of positive cells by the total number of ONL cells.

The signal intensity on retinal sections was quantified by Zen software version 2.3 (Zeiss). DAPI staining was used to identify the location of different retinal layers, and fluorescence intensity was manually recorded for each layer. For each count, fluorescence intensity was assessed at nine distinct positions. The values of all sections from the same animal were averaged before further analysis. Each experiment was independently performed at least three times using retinal explants obtained from different animals. No blinding was used during image acquisition and outcome assessment.

### RT-qPCR analysis

The total RNA was extracted from retinal explants using RNA extraction reagent (G3013, Servicebio, Wuhan, China), and cDNA was synthesized using SweScript All-in-One RT SuperMix for qPCR (G3337, Servicebio) following the manufacturers’ instructions. Quantitative real-time PCR was performed using 2×Universal Blue SYBR Green qPCR Master Mix (G3326, Servicebio). Relative RNA expression was quantified using the comparative 2^−ΔΔCt^ method and normalized to β-actin. Primer sequences are listed in Table 2.

**Table 2:** Primer list.

Gene	Forward primer	Reverse primer
*Ppargc1a*	GTACAACAATGAGCCTGCGAACA	TGCATCAAATGAGGGCAATCC
*Ppara*	TTTCACAAGTGCCTGTCTGTCG	TCTTCAGGTAGGCTTCGTGGAT
*Pparg*	GACCACTCGCATTCCTTTGACA	ATCGCACTTTGGTATTCTTGGA
*Nrf1*	TGCATCTCACCCTCCAAACC	GAAGCTGAGCCTGGGTCATT
*Esrra*	GCGGACGGCAGAAGTACAAAC	GCGACACCAGAGCGTTCACT
*Tfam*	GCTGATGGGTATGGAGAAGGAG	CCCTGAGCCGAATCATCCTTT
*Cpt1a*	GCACGAGGAAAAAATAAGCAATCT	TTGTCAAACCACCTGTCGAAAC
*Acox1*	CTGAAATCAAGAGAAGCGAGCC	CAGGTAGTAAAAGCCTTCAGCCC
*Fabp4*	GTGATGCCTTTGTGGGAACC	CCAGTTTGAAGGAAATCTCGGT
*Cd36*	GGAACTGTGGGCTATTGCT	CAACTTCCCTTTTGATTGTCTTCTC
*Actb*	GTGACGTTGACATCCGTAAAGA	GTAACAGTCCGCCTAGAAGCAC

### Statistical analysis and software use

Minimum animal and sample numbers were determined by an a priori power analysis based on effect sizes from previous explant studies [37, 38], performed using StatistikGuru (v1.96; https://www.statistikguru.de; accessed Oct. 2025). Animals were not allocated to experimental groups prior to sacrifice, and explants from both eyes of all animals were included without exclusion. Two-way comparisons were analyzed using Student’s *t-*test. Multiple comparisons were made using a one-way analysis of variance (ANOVA) test or two-way ANOVA with Dunnett’s multiple comparisons test. All tests were two-sided, normality was checked by Shapiro-Wilk testing, and variance homogeneity was evaluated using the Brown-Forsythe test. GraphPad Prism 10.6.1 software (GraphPad Software, San Diego, CA, USA) was used for statistical analysis and data visualization. Box-and-whisker plots were generated in OriginPro 2024b (OriginLab, Northampton, MA, USA). Data are presented as mean ± standard deviation (SD). The center values represent the mean, and the error bars represent SD. Levels of significance were as follows: ns, *p* > 0.05; *, *p* < 0.05; **, *p* < 0.01; ***, *p* < 0.001; ****, *p* < 0.0001. The figures were prepared using Adobe Photoshop 2020 (San Jose, CA, USA). Analysis of transcriptome datasets was carried out using Cytoscape 3.10.4 software (Cytoscape consortium) with the Molecular Complex Detection (MCODE) plugin [26] for module clustering and the cytoHubba plugin for hub gene identification and ranking [27].

## Supplementary information

Supplementary Information

## Data Availability

All data generated during this study are included in this published article and its Supplementary Materials Files. The GSE62020 *rd1* transcriptome dataset is available from NCBI´s GEO database (https://www.ncbi.nlm.nih.gov/geo/query/acc.cgi?acc=GSE62020; last accessed 07/2026).
