# Supplementary material for: PGC-1α and PPARs cooperatively mediate photoreceptor neuroprotection in rd1 mouse inherited retinal degeneration
Source: Cell Death Dis. 2026 Sep 14;17(1):797. doi: 10.1038/s41419-026-09254-3 (PMC13575171; doi:10.1038/s41419-026-09254-3)
Supplement: Supplementary file 1 [file 41419_2026_9254_MOESM1_ESM.pdf]

PGC-1α and PPARs cooperatively mediate photoreceptor neuroprotection in *rd1* mouse inherited retinal degeneration

Supplemental figures

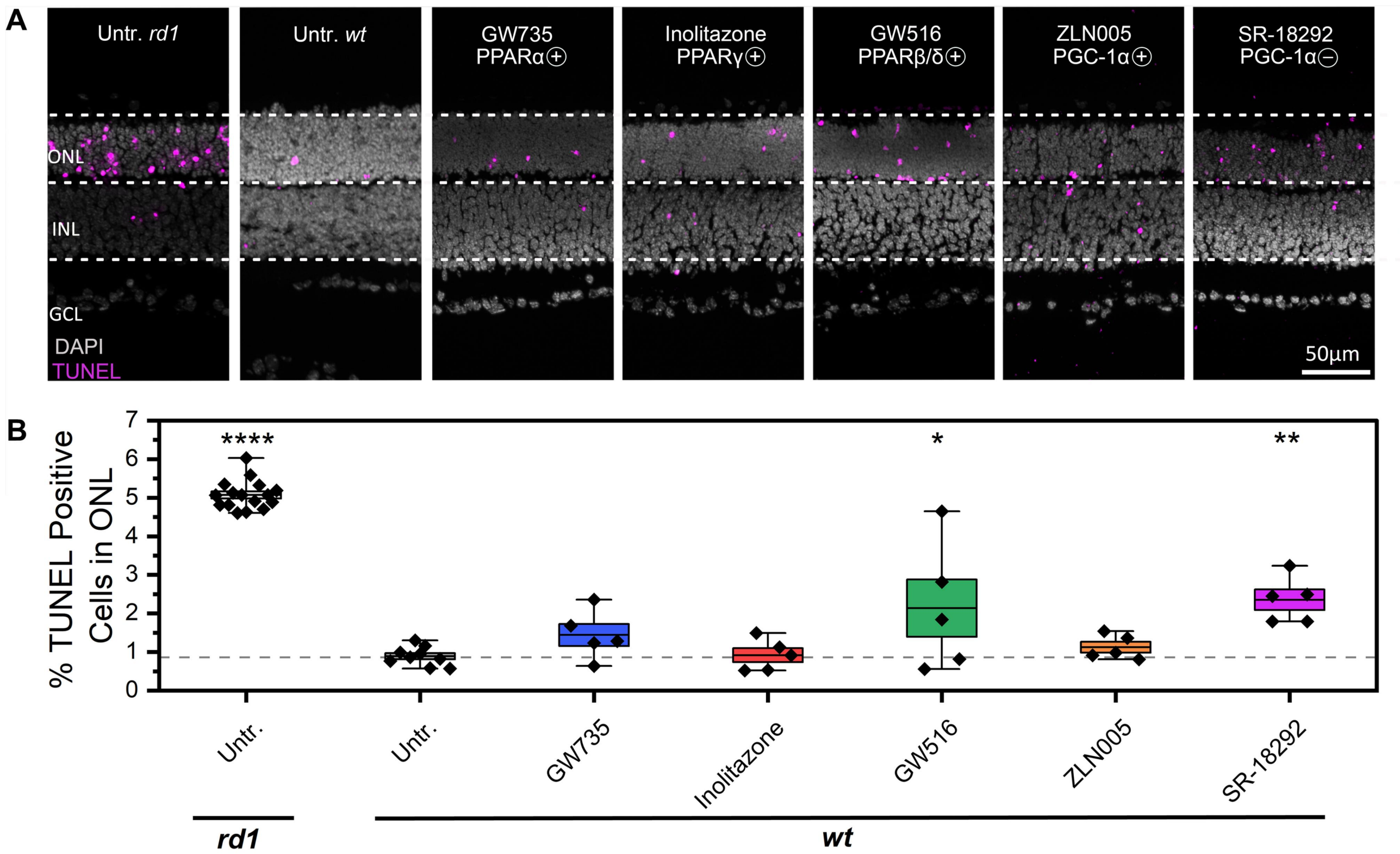

Figure S1. GW516 and SR-18292 increase wild-type photoreceptor cell death. A) TUNEL positive cells (magenta) in wild-type (*wt*) and *rd1* retinal (n=16) cultures. *wt* retina was left either untreated (Untr.) (n=9) or treated with PPARα agonist GW735 (n=5), PPARγ agonist Inolitazone (n=5), PPARβ/δ agonist GW516 (n=5), PGC-1α activator ZLN005 (n=5) and inhibitor SR-18292 (n=5). **B**) Box and whisker plot showing percentages of TUNEL positive cells in the outer nuclear layer (ONL). The grey dashed line designates *wt* untr. situation, data points below this threshold indicate protective effects, data points above suggest destructive effects. Statistical testing: Two-way ANOVA with Dunnett's multiple comparisons test performed between *rd1* and *wt* explant cultures. Error bars represent SD; ns= $p > 0.05$; * = $p < 0.05$; ** = $p < 0.01$; **** = $p < 0.0001$. INL = inner nuclear layer, GCL = ganglion cell layer; scale bar = 50 µm

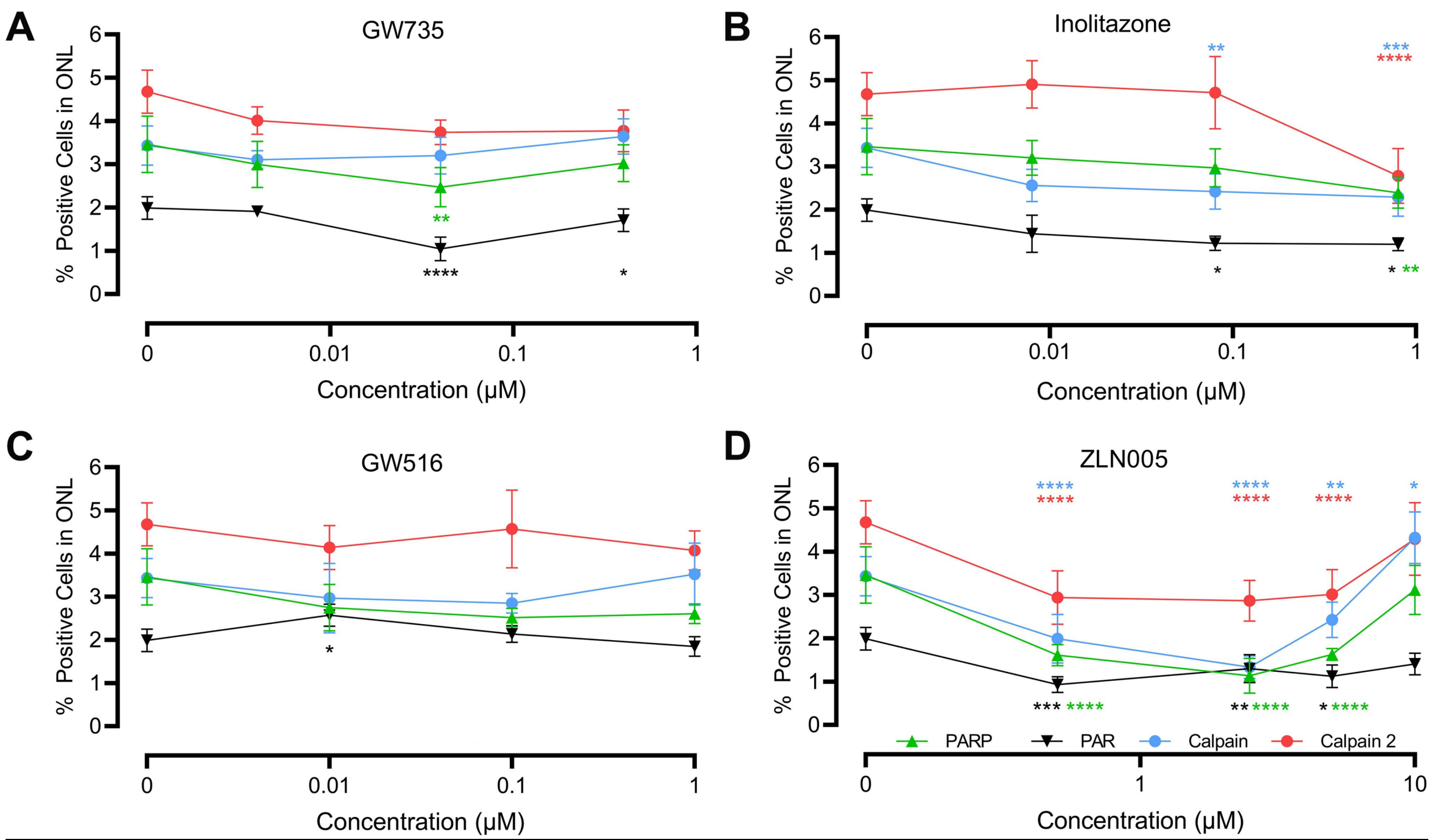

Figure S2. Dose-response curves for GW735, Inolitazone, GW516, and ZLN005. Markers assessed in the outer nuclear layer (ONL) of *rd1* retinal explant cultures were *in situ* PARP activity (green lines), accumulation of PAR (black), *in situ* calpain activity (blue), and activation of calpain-2 (red). **A**) In the ONL, at concentrations of 0.04 μM, the PPARα activator GW735 significantly decreased both the numbers of cells displaying PARP activity and PAR accumulation, at 0.4 μM it significantly reduced PAR accumulation. **B**) At concentrations of 0.8 μM, the PPARγ activator Inolitazone significantly reduced ONL PARP activity, PAR generation, calpain activity, and calpain 2 activation. 0.08 μM Inolitazone significantly reduced PAR generation and calpain activity. **C**) 0.01 μM of the PPAR β/δ activator GW516 significantly increased PAR production in the *rd1* ONL. **D**) The PGC-1α activator ZLN005, over a broad range of concentrations (0.5-5 μM), significantly reduced PARP activity, PAR generation, calpain activity, and calpain-2 activation in the ONL. Yet, at 10 μM, ZLN005 significantly increased calpain activity. The exact sample size (n) for each experimental group is provided in Table S5. Statistical significance was assessed using one-way ANOVA and Dunnett's multiple comparison test; significance levels: ns= $p > 0.05$; * = $p < 0.05$; ** = $p < 0.01$; *** = $p < 0.001$; **** = $p < 0.0001$; error bars represent SD.

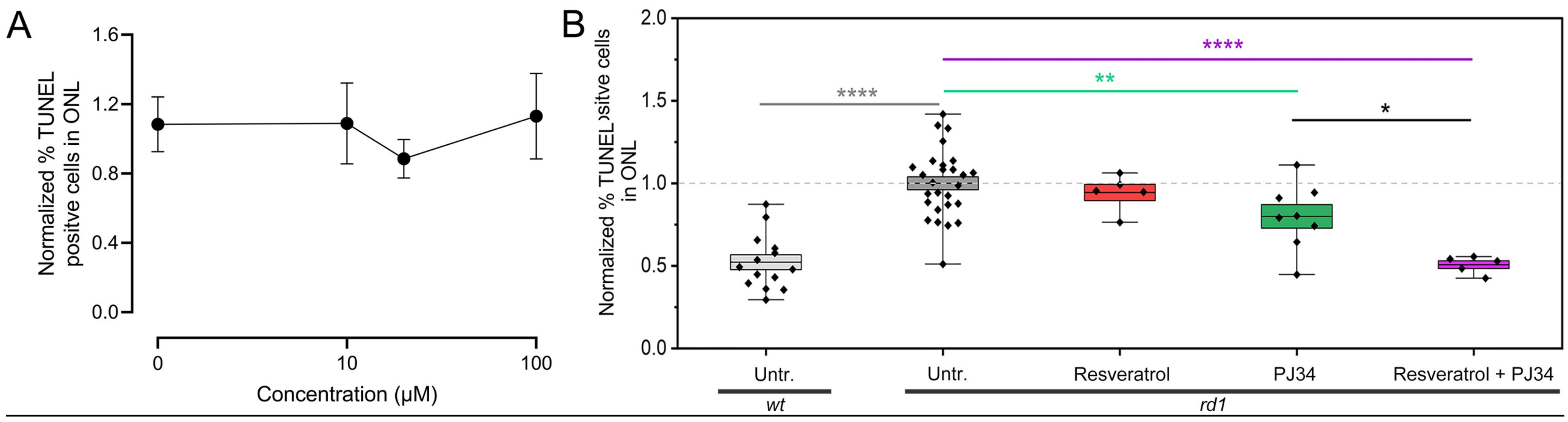

Figure S3. Sirtuin-1 dependent photoreceptor protection requires PARP inhibition. A) Dose-response curve for the sirtuin-1 agonist resveratrol (0 - 100 µM) in *rd1* mouse retinal explant cultures. Cell death in *rd1* outer nuclear layer (ONL) was assessed using the TUNEL assay, normalized to the untreated (Untr.) *rd1* situation. Resveratrol did not display significant *rd1* photoreceptor protection at any concentration tested. n=27 for untreated *rd1*, n=4, 5, and 5 for *rd1* explants treated with resveratrol at 10, 20, and 100 µM, respectively. **B)** Combination treatment with sirtuin-1 agonist and PARP antagonist. The grey dashed line designates *rd1* untr. situation, data points below this threshold indicate protective effects, data points above suggest destructive effects. In the ONL, at 20 µM, resveratrol did not decrease the number of TUNEL positive cells, while 6 µM of the PARP inhibitor PJ34 (n=8) significantly reduced the number of dying cells. However, 20 µM resveratrol combined with 6 µM PJ34 (n=5) further reduced TUNEL positive cells in *rd1* ONL. Statistical significance was assessed using one-way ANOVA and Dunnett's multiple comparison test in A, two-way ANOVA with Dunnett's multiple comparisons test in B; significance levels: ns= $p > 0.05$; * = $p < 0.05$; ** = $p < 0.01$; **** = $p < 0.0001$; error bars represent SD. Data was normalized by linear scaling according to the formula: $\chi_{\text{scaled}} = \chi \,/\, \overline{\chi}_{rd1,\text{Untr.}}$, here, χ is the TUNEL positive cells for the indicated condition, and $\overline{\chi}_{rd1,\ \text{Untr.}}$, is the mean of *rd1* untreated controls.

Supplemental Table 1. Quantification of TUNEL positive, dying cells in the outer nuclear layer (ONL).

A			Mean % positive cells in ONL	Standard deviation (SD)	Sample (n)	Dunnett's multiple comparisons test	Significance(*p*)
rd1		*rd1* Untr.	5.07	±0.38	16	*rd1* Untr. vs *wt* Untr.	*P* < 0.0001
	GW735	0.004 μM	4.69	±0.82	9	*rd1* Untr. vs 0.004 μM GW735	*P* > 0.05
		0.04μM	3.84	±0.37	9	*rd1* Untr. vs 0.04 μM GW735	*P* < 0.01
		0.4μM	4.23	±0.90	9	*rd1* Untr. vs 0.4 μM GW735	*P* > 0.05
	Inolitazone	0.008 μM	4.76	±0.81	12	*rd1* Untr. vs 0.008 μM Inolitazone	*P* > 0.05
		0.08 μM	4.21	±0.57	12	*rd1* Untr. vs 0.08 μM Inolitazone	*P* > 0.05
		0.8 μM	3.68	±0.54	12	*rd1* Untr. vs 0.8 μM Inolitazone	*P* < 0.001
	GW516	0.01 μM	4.68	±1.27	11	*rd1* Untr. vs 0.01 μM GW516	*P* > 0.05
		0.1 μM	4.40	±0.92	11	*rd1* Untr. vs 0.1 μM GW516	*P* > 0.05
		1 μM	4.30	±0.60	10	*rd1* Untr. vs 1 μM GW516	*P* > 0.05
	ZLN005	0.5 μM	3.24	±0.42	10	*rd1* Untr. vs 0.5 μM ZLN005	*P* < 0.0001
		2.5 μM	3.24	±0.83	9	*rd1* Untr. vs 2.5 μM ZLN005	*P* < 0.01
		5 μM	3.51	±0.49	7	*rd1* Untr. vs 5 μM ZLN005	*P* < 0.5
		10 μM	5.09	±0.93	5	*rd1* Untr. vs 10 μM ZLN005	*P* > 0.05
	SR-18292	1 μM	5.57	±0.47	6	*rd1* Untr. vs 1 μM SR-18292	*P* > 0.05
		5 μM	4.58	±0.15	6	*rd1* Untr. vs 5 μM SR-18292	*P* > 0.05
		10 μM	5.72	±1.25	6	*rd1* Untr. vs 10 μM SR-18292	*P* > 0.05
		20 μM	19.79	±2.16	6	*rd1* Untr. vs 20 μM SR-18292	*P* < 0.0001

B		Mean % positive cells in ONL	Standard deviation (SD)	Sample (n)	Dunnett's multiple comparisons test	Significance(*p*)
C3H	*wt* Untr.	0.89	±0.24	9	*wt* Untr. vs *rd1* Untr.	*P* < 0.0001
	GW735	1.44	±0.63	5	*wt* Untr. vs GW735	*P* > 0.05
	Inolitazone	0.92	±0.41	5	*wt* Untr. vs Inolitazone	*P* > 0.05
	GW516	2.32	±1.67	5	*wt* Untr. vs GW516	*P* < 0.05
	ZLN005	1.12	±0.32	5	*wt* Untr. vs ZLN005	*P* > 0.05
	SR-18292	2.35	±0.60	5	*wt* Untr. vs SR-18292	*P* < 0.01

Supplemental Table 2. Quantification of PARP activity and PAR positive cells in the outer nuclear layer (ONL).

A		Mean % positive cells in ONL	Standard deviation (SD)	Sample (n)	Dunnett's multiple comparisons test	Significance(*p*)
PARP activity	*wt* Untr.	0.19	±0.05	6	*rd1* Untr. vs *wt* Untr.	*P* < 0.0001
	rd1 Untr.	3.34	±0.26	10		
	GW735	2.47	±0.45	8	*rd1* Untr. vs GW735	*P* < 0.0001
	Inolitazone	2.40	±0.36	6	*rd1* Untr. vs Inolitazone	*P* < 0.0001
	GW516	3.01	±0.21	6	*rd1* Untr. vs GW516	*P* > 0.05
	ZLN005	1.61	±0.23	6	*rd1* Untr. vs ZLN005	*P* < 0.0001

B		Mean % positive cells in ONL	Standard deviation (SD)	Sample (n)	Dunnett's multiple comparisons test	Significance(*p*)
PAR	*wt* Untr.	0.37	±0.15	7	*rd1* Untr. vs *wt* Untr.	*P* < 0.0001
	rd1 Untr.	1.76	±0.44	11		
	GW735	1.04	±0.27	10	*rd1* Untr. vs GW735	*P* < 0.001
	Inolitazone	1.20	±0.15	6	*rd1* Untr. vs Inolitazone	*P* < 0.01
	GW516	1.85	±0.23	6	*rd1* Untr. vs GW516	*P* > 0.05
	ZLN005	1.10	±0.57	6	*rd1* Untr. vs ZLN005	*P* < 0.0001

Supplemental Table 3. Quantification of calpain activity, calpain-1 activation, and calpain-2 activation in the outer nuclear layer (ONL).

A		Mean % positive cells in ONL	Standard deviation (SD)	Sample (n)	Dunnett's multiple comparisons test	Significance(*p*)
Calpain activity	*wt* Untr.	0.31	±0.12	7	*rd1* Untr. vs *wt* Untr.	*P* < 0.0001
	rd1 Untr.	3.34	±0.46	16		
	GW735	3.09	±0.28	6	*rd1* Untr. vs GW735	*P* > 0.05
	Inolitazone	2.29	±0.44	5	*rd1* Untr. vs Inolitazone	*P* < 0.001
	GW516	3.52	±0.72	6	*rd1* Untr. vs GW516	*P* > 0.05
	ZLN005	1.99	±0.56	6	*rd1* Untr. vs ZLN005	*P* < 0.0001

B		Mean % positive cells in ONL	Standard deviation (SD)	Sample (n)	Dunnett's multiple comparisons test	Significance(*p*)
Calpain-1	*wt* Untr.	0.27	±0.05	6	*rd1* Untr. vs *wt* Untr.	*P* > 0.05
	rd1 Untr.	0.40	±0.05	10		
	GW735	0.41	±0.04	6	*rd1* Untr. vs GW735	*P* > 0.05
	Inolitazone	1.00	±0.24	6	*rd1* Untr. vs Inolitazone	*P* < 0.0001
	GW516	0.70	±0.12	6	*rd1* Untr. vs GW516	*P* < 0.01
	ZLN005	0.88	±0.23	6	*rd1* Untr. vs ZLN005	*P* < 0.0001

C		Mean % positive cells in ONL	Standard deviation (SD)	Sample (n)	Dunnett's multiple comparisons test	Significance(*p*)
Calpain-2	*wt* Untr.	0.51	±0.21	8	*rd1* Untr. vs *wt* Untr.	*P* < 0.0001
	rd1 Untr.	4.68	±0.50	9		
	GW735	4.08	±0.32	7	*rd1* Untr. vs GW735	*P* > 0.05
	Inolitazone	2.62	±0.56	5	*rd1* Untr. vs Inolitazone	*P* < 0.0001
	GW516	4.07	±0.45	5	*rd1* Untr. vs GW516	*P* > 0.05
	ZLN005	2.94	±0.62	6	*rd1* Untr. vs ZLN005	*P* < 0.0001

Supplemental Table 4. Effect of resveratrol or PJ34 on photoreceptor cell death (TUNEL assay) in the outer nuclear layer (ONL).

			Mean % positive cells in ONL	Standard deviation (SD)	Sample (n)	Dunnett's multiple comparisons test	Significance(*p*)
rd1	Untr.		3.55	±0.73	27	*rd1* Untr. vs *wt* Untr.	*P* < 0.0001
	Resveratrol	10 μM	3.74	±0.84	4	*rd1* Untr. vs 10 μM Resveratrol	*P* > 0.05
		20 μM	3.35	±0.39	5	*rd1* Untr. vs 20 μM Resveratrol	*P* > 0.05
		100 μM	3.87	±0.94	5	*rd1* Untr. vs 100 μM Resveratrol	*P* > 0.05
	PJ34		2.83	±0.71	8	*rd1* Untr. vs PJ34	*P* < 0.01
	Resveratrol + PJ34		1.80	±0.19	5	*rd1* Untr. vs Resveratrol + PJ34	*P* < 0.0001
C3H	Untr.		1.85	±0.59	14	*wt* Untr. vs *rd1* Untr.	*P* < 0.0001

Supplemental Table 5. Quantification of PARP activity, PAR-positive cells, calpain activity, and calpain-2 activation in the outer nuclear layer (ONL) after dose-response treatment with GW735, Inolitazone, GW516, and ZLN005.

A			Mean % positive cells in ONL	Standard deviation (SD)	Sample (n)	Dunnett's multiple comparisons test	Significance(*p*)
GW735	PARP activity	*rd1* Untr.	3.34	±0.26	10		
		0.004 μM	3.00	±0.53	5	*rd1* Untr. vs 0.004 μM GW735	*P* > 0.05
		0.04μM	2.47	±0.45	8	*rd1* Untr. vs 0.04 μM GW735	*P* < 0.01
		0.4μM	3.02	±0.42	8	*rd1* Untr. vs 0.4 μM GW735	*P* > 0.05
	PAR	*rd1* Untr.	1.76	±0.44	11		
		0.004 μM	1.91	±0.06	5	*rd1* Untr. vs 0.004 μM GW735	*P* > 0.05
		0.04μM	1.04	±0.27	10	*rd1* Untr. vs 0.04 μM GW735	*P* < 0.0001
		0.4μM	1.70	±0.26	5	*rd1* Untr. vs 0.4 μM GW735	*P* < 0.05
	Calpain activity	*rd1* Untr.	3.34	±0.46	16		
		0.004 μM	3.11	±0.21	4	*rd1* Untr. vs 0.004 μM GW735	*P* > 0.05
		0.04μM	3.09	±0.28	8	*rd1* Untr. vs 0.04 μM GW735	*P* > 0.05
		0.4μM	3.64	±0.41	7	*rd1* Untr. vs 0.4 μM GW735	*P* > 0.05
	Calpain-2	*rd1* Untr.	4.68	±0.50	9		
		0.004 μM	4.01	±0.32	4	*rd1* Untr. vs 0.004 μM GW735	*P* > 0.05
		0.04μM	4.08	±0.32	7	*rd1* Untr. vs 0.04 μM GW735	*P* > 0.05
		0.4μM	3.77	±0.48	5	*rd1* Untr. vs 0.4 μM GW735	*P* > 0.05

B			Mean % positive cells in ONL	Standard deviation (SD)	Sample (n)	Dunnett's multiple comparisons test	Significance(*p*)
Inolitazone	PARP activity	*rd1* Untr.	3.34	±0.26	10		
		0.008 μM	3.20	±0.40	6	*rd1* Untr. vs 0.008 μM Inolitazone	*P* > 0.05
		0.08 μM	2.97	±0.44	5	*rd1* Untr. vs 0.08 μM Inolitazone	*P* > 0.05
		0.8 μM	2.40	±0.36	6	*rd1* Untr. vs 0.8 μM Inolitazone	*P* < 0.01
	PAR	*rd1* Untr.	1.76	±0.44	11		
		0.008 μM	1.44	±0.43	6	*rd1* Untr. vs 0.008 μM Inolitazone	*P* > 0.05
		0.08 μM	1.22	±0.16	6	*rd1* Untr. vs 0.08 μM Inolitazone	*P* < 0.05
		0.8 μM	1.20	±0.15	6	*rd1* Untr. vs 0.8 μM Inolitazone	*P* < 0.05
	Calpain activity	*rd1* Untr.	3.34	±0.46	16		
		0.008 μM	2.56	±0.37	6	*rd1* Untr. vs 0.008 μM Inolitazone	*P* > 0.05
		0.08 μM	2.42	±0.41	5	*rd1* Untr. vs 0.08 μM Inolitazone	*P* < 0.01
		0.8 μM	2.29	±0.44	5	*rd1* Untr. vs 0.8 μM Inolitazone	*P* < 0.001
	Calpain-2	*rd1* Untr.	4.68	±0.50	9		
		0.008 μM	4.90	±0.55	6	*rd1* Untr. vs 0.008 μM Inolitazone	*P* > 0.05
		0.08 μM	4.71	±0.84	6	*rd1* Untr. vs 0.08 μM Inolitazone	*P* > 0.05
		0.8 μM	2.62	±0.56	5	*rd1* Untr. vs 0.8 μM Inolitazone	*P* < 0.0001

C			Mean % positive cells in ONL	Standard deviation (SD)	Sample (n)	Dunnett's multiple comparisons test	Significance(*p*)
GW516	PARP activity	*rd1* Untr.	3.34	±0.26	10		
		0.01 μM	2.75	±0.54	4	*rd1* Untr. vs 0.01 μM GW516	*P* > 0.05
		0.1 μM	2.51	±0.22	4	*rd1* Untr. vs 0.1 μM GW516	*P* > 0.05
		1 μM	3.01	±0.21	6	*rd1* Untr. Vs 1 μM GW516	*P* > 0.05
	PAR	*rd1* Untr.	1.76	±0.44	11		
		0.01 μM	2.57	±0.26	6	*rd1* Untr. vs 0.01 μM GW516	*P* < 0.05
		0.1 μM	2.14	±0.20	6	*rd1* Untr. vs 0.1 μM GW516	*P* > 0.05
		1 μM	1.85	±0.23	6	*rd1* Untr. Vs 1 μM GW516	*P* > 0.05
	Calpain	*rd1* Untr.	3.34	±0.46	16		
		0.01 μM	2.97	±0.81	4	*rd1* Untr. vs 0.01 μM GW516	*P* > 0.05

	activity	0.1 μM	2.85	±0.23	5	*rd1* Untr. vs 0.1 μM GW516	*P* > 0.05
		1 μM	3.52	±0.72	6	*rd1* Untr. Vs 1 μM GW516	*P* > 0.05
	Calpain-2	*rd1* Untr.	4.68	±0.50	9		
		0.01 μM	4.14	±0.51	6	*rd1* Untr. vs 0.01 μM GW516	*P* > 0.05
		0.1 μM	4.57	±0.90	6	*rd1* Untr. vs 0.1 μM GW516	*P* > 0.05
		1 μM	4.07	±0.45	5	*rd1* Untr. Vs 1 μM GW516	*P* > 0.05

**D**			Mean % positive cells in ONL	Standard deviation (SD)	Sample (n)	Dunnett's multiple comparisons test	Significance(*p*)
ZLN005	PARP activity	*rd1* Untr.	3.34	±0.26	10		
		0.5 μM	1.61	±0.23	6	*rd1* Untr. vs 0.5 μM ZLN005	*P* < 0.0001
		2.5 μM	1.13	±0.40	4	*rd1* Untr. vs 2.5 μM ZLN005	*P* < 0.0001
		5 μM	1.63	±0.14	5	*rd1* Untr. vs 5 μM ZLN005	*P* < 0.0001
		10 μM	3.11	±0.57	6	*rd1* Untr. vs 10 μM ZLN005	*P* > 0.05
	PAR	*rd1* Untr.	1.76	±0.44	11		
		0.5 μM	1.10	±0.57	6	*rd1* Untr. vs 0.5 μM ZLN005	*P* < 0.001
		2.5 μM	1.30	±0.33	6	*rd1* Untr. vs 2.5 μM ZLN005	*P* < 0.01
		5 μM	1.13	±0.26	6	*rd1* Untr. vs 5 μM ZLN005	*P* < 0.05
		10 μM	1.41	±0.25	6	*rd1* Untr. vs 10 μM ZLN005	*P* > 0.05
	Calpain activity	*rd1* Untr.	3.34	±0.46	16		
		0.5 μM	1.99	±0.56	6	*rd1* Untr. vs 0.5 μM ZLN005	*P* < 0.0001
		2.5 μM	1.33	±0.26	4	*rd1* Untr. vs 2.5 μM ZLN005	*P* < 0.0001
		5 μM	2.43	±0.41	5	*rd1* Untr. vs 5 μM ZLN005	*P* < 0.01
		10 μM	4.32	±0.60	4	*rd1* Untr. vs 10 μM ZLN005	*P* < 0.05
	Calpain-2	*rd1* Untr.	4.68	±0.50	9		
		0.5 μM	2.94	±0.62	6	*rd1* Untr. vs 0.5 μM ZLN005	*P* < 0.0001
		2.5 μM	2.87	±0.47	6	*rd1* Untr. vs 2.5 μM ZLN005	*P* < 0.0001
		5 μM	3.01	±0.57	6	*rd1* Untr. vs 5 μM ZLN005	*P* < 0.0001
		10 μM	4.30	±0.84	6	*rd1* Untr. vs 10 μM ZLN005	*P* > 0.05
